# The Essential Component in DNA-Based Information Storage System: Robust Error-Tolerating Module

**DOI:** 10.3389/fbioe.2014.00049

**Published:** 2014-11-06

**Authors:** Aldrin Kay-Yuen Yim, Allen Chi-Shing Yu, Jing-Woei Li, Ada In-Chun Wong, Jacky F. C. Loo, King Ming Chan, S. K. Kong, Kevin Y. Yip, Ting-Fung Chan

**Affiliations:** ^1^School of Life Sciences, The Chinese University of Hong Kong, Hong Kong, China; ^2^Hong Kong Bioinformatics Centre, The Chinese University of Hong Kong, Hong Kong, China; ^3^State Key Laboratory of Argobiotechnology, The Chinese University of Hong Kong, Hong Kong, China; ^4^Department of Computer Science and Engineering, The Chinese University of Hong Kong, Hong Kong, China

**Keywords:** DNA-based information storage, error-tolerating module, DNA-based computational process, synthetic biology, biocomputing

## Abstract

The size of digital data is ever increasing and is expected to grow to 40,000 EB by 2020, yet the estimated global information storage capacity in 2011 is <300 EB, indicating that most of the data are transient. DNA, as a very stable nano-molecule, is an ideal massive storage device for long-term data archive. The two most notable illustrations are from Church et al. and Goldman et al., whose approaches are well-optimized for most sequencing platforms – short synthesized DNA fragments without homopolymer. Here, we suggested improvements on error handling methodology that could enable the integration of DNA-based computational process, e.g., algorithms based on self-assembly of DNA. As a proof of concept, a picture of size 438 bytes was encoded to DNA with low-density parity-check error-correction code. We salvaged a significant portion of sequencing reads with mutations generated during DNA synthesis and sequencing and successfully reconstructed the entire picture. A modular-based programing framework – DNAcodec with an eXtensible Markup Language-based data format was also introduced. Our experiments demonstrated the practicability of long DNA message recovery with high error tolerance, which opens the field to biocomputing and synthetic biology.

## Introduction

Various research teams have studied the total amount of data generated, stored, and consumed in the world, and although the underlying assumptions vary and lead to differences in results, all of them are expecting exponential growth in the years ahead. In 2012, the International Data Corporation^1^ and EMC Corporation^2^ estimated the size of digital data as 2,837 EB (Exabytes) and a doubling time of roughly 2 years – the size of digital information will grow to 40,000 EB by 2020. Yet, in 2011 IDC estimated that the global information storage capacity is 264 EB, while Hilbert et al. estimated that the global information storage capacity is 295 EB, both indicating that most of the data generated these days are transient – physically impossible to be stored. One example in the field of Bioinformatics would be the sequence read archive (SRA), a raw data repository of sequencing data that is run by the INSDC partners^3^, where the original image data from next-generation sequencing (NGS) platforms were discarded, and the repository retained only the sequencing reads. It is estimated that this could achieve a 200- to 500-fold reduction in data size when compared to raw data with image information (Hsi-Yang Fritz et al., 2011). However, once the raw image data were discarded, the research group could no longer repeat the base-calling step from the raw image data when uncertainty was present during the process. This would also hindered the development of new base-calling algorithms (Massingham and Goldman, 2012) as most research groups would only have access to compressed sequencing reads from SRA. Big data drive huge demand for storage capacity; development of new storage device requires high data density with respect to physical size in order to maximize storage efficiency. As a nano-molecule with well-established synthesis and sequencing technologies developed, DNA is an ideal massive information storage device for data archive.

Baum first introduced information storage in DNA in 1995 (Baum, 1995), with a proposed content addressable memory structure that enables rapid searching in data. In 1999, Clelland et al. further developed a DNA-based, doubly steganographic technique (Clelland et al., 1999) for encoding secret messages in DNA. In 2001, Bancroft et al. had listed three reasons (Bancroft et al., 2001) that makes DNA desirable for long-term information storage: (1) with an extreme stability (Paabo et al., 1988; Vreeland et al., 2000), DNA has stood the informational “test of time” during the billions of years since life emerged; (2) DNA as genetic material, techniques in storage, synthesis, and sequencing would continually be developed and remain central to technological civilization; (3) DNA as a storage medium would allow an extensive informational redundancy as each segment of information could be stored repeatedly. Both Baum and Bancroft proposed a similar data structure for information storage in DNA that involves common flanking forward (F) and reverse (R) PCR amplification primers and a unique sequencing primer together with the information segment. Their research set a solid framework for further development of information storage in DNA.

Advancement in using DNA as an information storage medium continues, both experimentally (Pak Chung Wong, 2003; Kashiwamura et al., 2005; Yachie et al., 2007) and algorithmically (Ailenberg and Rotstein, 2009). Yet, practical and large-scale implementation remains unfavorable due to technological limitations on robust DNA synthesis and sequencing technology. It was not until 2012 that such a feat was achieved, when Church et al. (2012) encoded the book *Regenisis*, synthesized it into DNA, and adopted the NGS technology to decipher the DNA information. Their group also created the “one bit per base” coding system. In 2013, Goldman et al. (2013) inserted at present the largest piece of information (size of 757 kb) into the DNA-based storage system. They further improved the encoding scheme by utilizing the purpose-designed Huffman Code together with a base-3 to DNA encoding system (“trits” 0, 1, and 2). Each of the 256 possible bytes of ASCII code was represented by five or six trits, and the Huffman code was provided in their Supplementary file – View_huff3.cd.new. With this specific coding scheme, the subsequent DNA base is defined by the previous DNA base, hence eliminating the existence of consecutive identical DNA bases – the homopolymer issue. They also introduced a fourfold redundancy scheme with 75 bases overlapping for each DNA information block being synthesized to ensure data integrity. It is now clear that obstacles toward practical usage of DNA as an information storage medium are gradually being resolved. Moreover, advancement in DNA synthesis such as the transition to array-based oligos is also expected to lower the cost of synthesis by three to five orders of magnitude, on par with the cost of oligo pools ($1 per 10^3^–10^5^ bp) (Kosuri and Church, 2014). Both Church’s and Goldman’s research have set the infrastructure of DNA-based information storage. To further improve the information encoding scheme in terms of data integrity and error handling, we believe the introduction of error-correction model would be essential along the development of a DNA-based information storage system.

## Limitations on Currently Proposed Storage Systems

There are limitations on the existing DNA-based information storage system. Church’s method involved the “one bit per base” coding system with base “A/C” for zero and “G/T” for one. Yet, they also identified a relatively high error rate and low sequencing coverage due to the homopolymer issue and the presence of repetitive sequences. For Goldman’s approach, although the homopolymer issue associated with DNA sequencing could be eliminated by their proposed base-3 encoding methodology, it is being transferred to short tandem repeats that may cause assembly error – for example, a consecutive series of zero bits would give repeated sequence of “CGTA.” Both Church and Goldman had chosen to synthesize short DNA information block for encoding (Church’s work: 115 bp; Goldman’s work: 117 bp), we believe that there are three major reasons: (1) easiness in implementation as no downstream *de novo* assembly is required, hence no assembly error has to be addressed; (2) higher throughput in DNA synthesis that would always cover the loss of information space due to the length limitation on DNA information block; (3) the proposed length of DNA information block could be scaled up as the DNA sequencing and synthesis technologies mature. However, the advantage of using DNA-based information storage is not only limited to its high data density with respect to its physical size but also the sophisticated manipulation techniques used in other DNA-based computational process such as recombination (Bonnet et al., 2012) and the self-assembly nature of DNA (Mao et al., 2000; Treangen and Salzberg, 2012), which in turn would require a better error-tolerating information encoding system and longer DNA sequences to be synthesized.

In this study, we encoded the logo of our university into DNA bases using advanced data compression and error-correction models, together with a non-overlapping DNA synthesis approach for six DNA information blocks, each with length up to 720 bp – 6.2× longer than Goldman’s work. We were able to recover the picture successfully without introducing any noise.

## Proof of Concepts for Compression and Error-Correction Models

**Figure 1:**
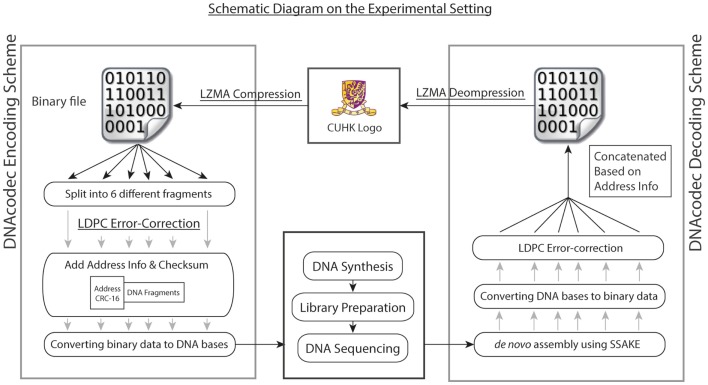
**Overall experimental design is shown**. As a proof of concept, the logo of our university in BMP format was first compressed with Lempel-Ziv-Markov chain algorithm (LZMA). The binary file after compression was then split into six different fragments, each contained different sections of the binary file without overlapping. Each fragment was then embedded with low-density parity-check (LDPC) error-correction code, and the header containing the address information was then added in front of the DNA fragment. Each fragment, together with the header information, was first converted into quaternary numerical system, and further encoded into DNA bases (termed as DNA information block) by simple base conversion (A: 0, T: 1, C: 2, G: 3). The six DNA information blocks were then synthesized, each with a total size of 720 bp. To decode the information, the pool of synthesized DNA was then subjected to high-throughput sequencing using Illumina GA IIx platform. Upon quality trimming on raw reads, *de novo* assembly was performed. The six DNA information block could be recovered by using overlay-consensus approach, and the DNA bases were then converted back to a quaternary numerical system and further into binary format. LDPC code was then used to correct for the random mutations generated during the DNA synthesis and sequencing process. The six fragments were then concatenated based on the address information and decompressed using LZMA. The image could be recovered completely.

To better illustrate the practicality of using advanced data compression and error-correction models in DNA-based information storage, we have performed both *in vitro* and *in silico* experiments. For our *in vitro* experiment, a picture of size 30 × 20 pixel (438 bytes) in BMP format was first compressed with Lempel-Ziv-Markov chain algorithm (LZMA)^4^, which is a lossless data compression algorithm using a dictionary compression approach, with huge dictionary sizes for repeated sequences (Solomon, 2006). The binary file after compression was then sub-divided into six fragments without overlapping, and each fragment was then embedded with low-density parity-check (LDPC) error-correction code in view of possible mutations arisen during the DNA synthesis and sequencing process. LDPC is a linear error-correction code for transmitting a message over a noisy transmission channel (Gallager, 1963). One important feature is that LDPC codes are capacity-approaching codes, which enables data transfer at a near Shannon Limit Performance channel (Neal, 1996). The header containing the address information with respect to the fragment was then added in the front. Each fragment was then converted to a quaternary numerical system, and further encoded into DNA bases (termed as DNA information block) by simple base conversion (A: 0, T: 1, C: 2, G: 3). The schematic diagram summarizing our experimental design is shown in Figure 1.

The six DNA information blocks were then synthesized, each with a total size of 720 bp. To decode the information in DNA, the pool of synthesized DNA was then subjected to high-throughput sequencing using Illumina GA IIx platform, generating 1.92 M read-pairs of 76 bp with an insert size of 200 bp (NCBI Short Read Archive accession: SRR726231). Quality trimming on raw reads was performed with a minimum quality score of 32. *De novo* assembly by overlay-consensus approach was done by using SSAKE v3.8 (Warren et al., 2007) (parameters: −w 1; −m 50; insert size 200 bp; header sequences were used as seed for extension), and the *de novo* assembly results were manually inspected by IGV (Thorvaldsdottir et al., 2013). Based on re-mapping results of reads using Novoalign v3.00.05^5^, 96.32% (1.85 M out of 1.92 M reads) were used in the assembly process and 85.82% (1.64 M out of 1.92 M reads) of reads were properly paired. Each DNA information block was then converted back to binary fragments, followed by LDPC error correction to correct for the random mutations generated during the DNA synthesis and sequencing process. The six fragments were then concatenated based on the address information and decompressed using LZMA. The image could be fully recovered.

**Figure 2:**
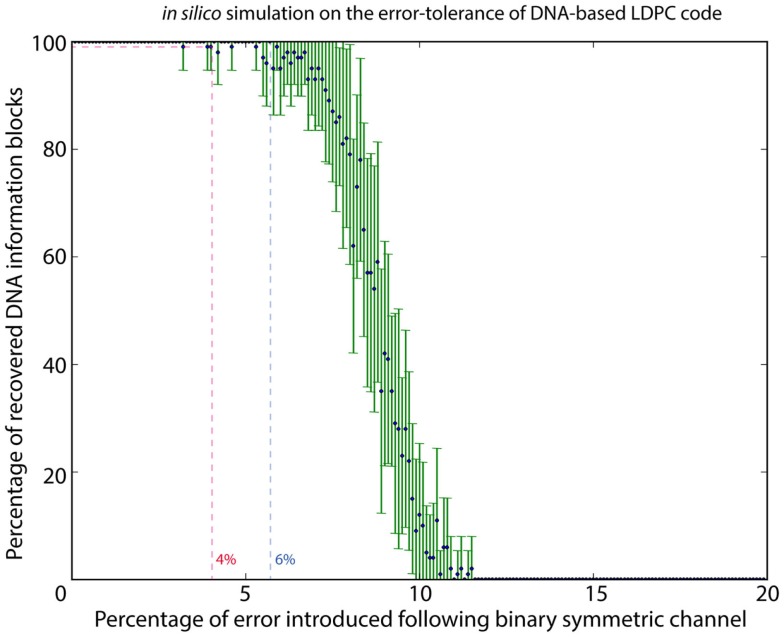
***In silico* simulation to characterize the error tolerance of DNA-based LDPC code**. By using a binary symmetric channel as the error model, a range of error rates from 0 to 20% was used with a stepping increment of 0.1%. Random errors simulating substitution were introduced in each step at its specific error rate percentage, and each step was iterated for 20 times. DNA-based LDPC model could withstand an error rate up to 4%.

To estimate the error rate associated with DNA synthesis and sequencing, 1.92 M reads were mapped to the original sequence-encoded DNA sequence of the logo using Novoalign v3.00.05; 15.91% of the reads showed mismatches in the form of substitution or indel. At higher resolution of bases, 0.19% of bases showed a substitution, with 0.01% of bases were annotated as insertion and 0.38% of bases being deleted. Although the DNA-based LDPC model could correct all the errors and completely recover the image file, we also need to know the maximum error rate that the model could withstand. We therefore performed *in silico* analysis to determine the effect of different error rates on information recovery using the DNA-based LDPC model. With the error model following the binary symmetric channel, a range of error rates from 0 to 20% was used with a stepping increment of 0.1%. Random errors simulating substitution of DNA bases were introduced at each increment, and each increment was repeated for 20 times. The result in Figure 2 shows that DNA-based LDPC model could withstand an error rate of up to 4%.

One observation is that the sequencing fold coverage for each six fragments is not uniform, with a range from 104× to 104,938× coverage (Table S1 in Supplementary Material). This could be due to bias in DNA library preparation during the sequencing step. Although all six DNA information blocks could be assembled, it is also important to characterize the minimum fold coverage necessary for the DNA information block to be *de novo* assembled. Therefore, the third DNA information block, which had fold coverage of 104,938×, was randomly down-sampled to 1–500× coverage. *De novo* assembly was performed iteratively 100 times using SSAKE v3.8 from 1× to 500× coverage with a stepping increment of 1× coverage, and the assembled sequence was then compared with the true sequence using BLAST. The average percentage identity at each step increment is shown in Figure S1 in Supplementary Material. In order to reach an average percentage identity over 97%, a 90× coverage is essential for the *de novo* assembly.

## Discussion

Rapid development of using DNA as an information storage medium is expected; hence, it is important to establish a framework that is solid yet has enough elasticity to accommodate modifications from different groups. In this study, we have developed a modular data encoding/decoding program – DNAcodec, which is extensible via the eXtensible Markup Language (XML)-based data interchange format. The choice of XML enables high degree of interoperability with existing programs, tool-chains, and development tools. The code can be obtained at Github^6^, and the description of the program as well as XML-based data structure was documented in detail.

In this experiment, while it is only at a miniscule scale setting, we have successfully recovered the image without any noise introduced and demonstrated the effectiveness of an advanced error-correction model in handling both synthesis and sequencing errors during the information decoding process. *In vivo* experiment showed that 96% of the reads were used for the *de novo* assembly process, with 85% of the reads were properly paired. During DNA synthesis and sequencing, 0.58% of the bases experienced mutations including substitution and indel, and they span across 16% of the total reads. If the filtering criteria from Goldman et al. or Church et al. were adopted, at least 16% of the reads would have to be discarded in this simple experiment. Indeed in Goldman’s study, they adopted even more stringent filtering criteria, which removed up to 37% of all reads (as shown in Table S3 in Supplementary Material) and retained only 63% for information decoding. In addition, they used an extensive overlapping approach that further reduced the data capacity to within 63% of reads. In this experiment, LDPC was used because of its reliability, and because it is well-adapted to numerous applications. LDPC is an open-source software and could be included into the module-based pipeline. Overall, we believe a good error-correction model, not necessarily using LDPC, would be essential for reducing the throughput and further lowering the cost of data storage.

It is noteworthy in this experiment that even though insertions/deletions (indel) were identified by reference-based mapping of sequencing reads, no indel was observed in the *de novo* assembled DNA information block. This indicates that majority voting during the *de novo* assembly process was enough to correct for indels in our experiment. In our system, we made a reasonable assumption that the distribution of error associated with sequencing and synthesis is random, such that majority vote should be able to serve as a confident measurement. With the extensive coverage of DNA information blocks by sequencing reads, the positions of all potential indel sites, when present as sequencing noise, could always be identified by majority voting. Also, it is unlikely that errors associated with sequencing and synthesis would accumulate at one position and becomes the majority. We therefore performed *in silico* analysis with the error model following the binary symmetric channel, simulating the substitution error, and the results in Figure 2 shows that our proposed model could withstand an error rate of up to 4%.

DNA-based storage medium has very different characteristics from that of traditional data storage media. For example, data manipulation such as distributing copies would require DNA replication that may generate mutations. Future work in the area of synthetic biology may also integrate biocomputing concepts with DNA as information storage devices. *In silico* down-sampling analysis showed that with the read length of 76 bp and insert size of 200 bp, one would be able to retrieve a DNA information block of length 720 bp with a minimum coverage of 90× using a *de novo* approach. A 90× coverage for a 720 bp sequence is insignificant when compared to the throughput of current sequencing platform. Indeed with the advancement of sequencing technology such as PacBio RS II, long read lengths of over 10 k bases is commercially available, yet with relatively high associated sequencing error. It is expected that a robust error-tolerating storage system would be critical for the development of DNA-based storage system. With the achievement of reaching an information storage density of ~2.2 PB/g by Goldman et al., it is expected that by incorporating our proposed error-tolerating modules, the information storage density could further increase by incorporating a lower or even no redundancy storage scheme. Our proposed components not only facilitate regular data handling with both synthesis and sequencing error tolerance but also demonstrate the practicability of retrieving information from long DNA information blocks through *de novo* assembly, which would allow the implementation of other DNA-based computational algorithms in the future.

## Author Contributions

Ting-Fung Chan, Kevin Y. Yip, S. K. Kong, and King Ming Chan supervised the project. Aldrin Kay-Yuen Yim, Jing-Woei Li, and Allen Chi-Shing Yu originated the idea and prepared the manuscript. Aldrin Kay-Yuen Yim and Jing-Woei Li performed the bioinformatics analysis. Aldrin Kay-Yuen Yim, Allen Chi-Shing Yu, Jing-Woei Li, Ada In-Chun Wong, and Jacky F. C. Loo prepared the DNA for sequencing. Allen Chi-Shing Yu devised the modular data encoding/decoding program – DNAcodec. Aldrin Kay-Yuen Yim, Allen Chi-Shing Yu, and Jing-Woei Li contributed equally in this work. All authors read and approved the final manuscript.

## Conflict of Interest Statement

The authors declare that the research was conducted in the absence of any commercial or financial relationships that could be construed as a potential conflict of interest.

## Supplementary Material

The Supplementary Material for this article can be found online at http://www.frontiersin.org/Journal/10.3389/fbioe.2014.00049/abstract

## Footnotes

^1^http://www.idc.com

^2^http://www.emc.com

^3^http://www.insdc.org

^4^http://www.7-zip.org/sdk.html

^5^http://www.novocraft.com/

^6^http://github.com/a113n/DNAcodec
