# Supplementary material for: The Essential Component in DNA-Based Information Storage System: Robust Error-Tolerating Module
Source: Front Bioeng Biotechnol. 2014 Nov 6;2:49. doi: 10.3389/fbioe.2014.00049 (PMC4222239; doi:10.3389/fbioe.2014.00049)
Supplement: Supplementary file 1 [file Data_Sheet_1.DOCX]

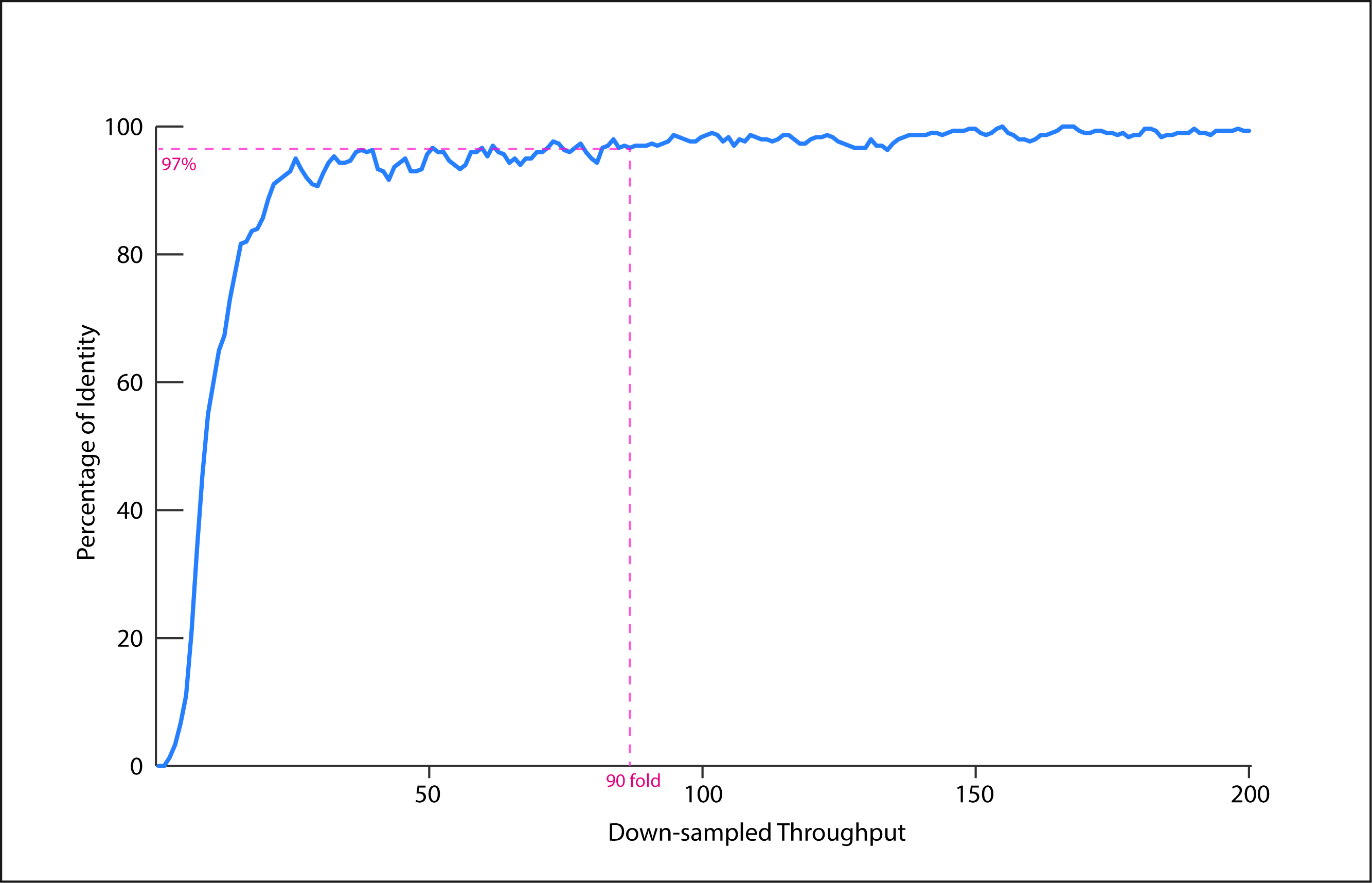


Figure S1. Estimation of the minimum coverage necessary for data recovery by down-sampling the third DNA information blocks from 1x to 200x coverage for *de novo* assembly.

Table S1. Sequencing coverage for 6 DNA information block synthesized in this experiment

| **DNA Information Block** | **Sequencing Fold Coverage** |
| --- | --- |
| 1 | 48013.62 |
| 2 | 7991.01 |
| 3 | 104938.25 |
| 4 | 104.67 |
| 5 | 980.77 |
| 6 | 195.47 |
